# Selective Sweep Analysis in the Genomes of the *91-R* and *91-C Drosophila melanogaster* Strains Reveals Few of the ‘Usual Suspects’ in Dichlorodiphenyltrichloroethane (DDT) Resistance

**DOI:** 10.1371/journal.pone.0123066

**Published:** 2015-03-31

**Authors:** Laura D. Steele, Brad Coates, M. Carmen Valero, Weilin Sun, Keon Mook Seong, William M. Muir, John M. Clark, Barry R. Pittendrigh

**Affiliations:** 1 Department of Entomology, University of Illinois, Urbana-Champaign, Illinois, United States of America; 2 United States Department of Agriculture, Agricultural Research Service, Corn Insects and Crop Genetics Research Unit, Iowa State University, Ames, Iowa, United States of America; 3 Department of Animal Sciences, Purdue University, West Lafayette, Indiana, United States of America; 4 Department of Veterinary & Animal Science, University of Massachusetts, Amherst, Massachusetts, United States of America; Universidade Federal do Rio de Janeiro, BRAZIL

## Abstract

Adaptation of insect phenotypes for survival after exposure to xenobiotics can result from selection at multiple loci with additive genetic effects. To the authors’ knowledge, no selective sweep analysis has been performed to identify such loci in highly dichlorodiphenyltrichloroethane (DDT) resistant insects. Here we compared a highly DDT resistant phenotype in the *Drosophila melanogaster* (*Drosophila) 91-R* strain to the DDT susceptible *91-C* strain, both of common origin. Whole genome re-sequencing data from pools of individuals was generated separately for *91-R* and *91-C*, and mapped to the reference *Drosophila* genome assembly (v. 5.72). Thirteen major and three minor effect chromosome intervals with reduced nucleotide diversity (*π*) were identified only in the *91-R* population. Estimates of Tajima's D (*D*) showed corresponding evidence of directional selection in these same genome regions of *91-R*, however, no similar reductions in π or *D* estimates were detected in *91-C*. An overabundance of non-synonymous proteins coding to synonymous changes were identified in putative open reading frames associated with *91-R*. Except for *NinaC* and *Cyp4g1*, none of the identified genes were the ‘usual suspects’ previously observed to be associated with DDT resistance. Additionally, up-regulated ATP-binding cassette transporters have been previously associated with DDT resistance; however, here we identified a structurally altered *MDR49* candidate resistance gene. The remaining fourteen genes have not previously been shown to be associated with DDT resistance. These results suggest hitherto unknown mechanisms of DDT resistance, most of which have been overlooked in previous transcriptional studies, with some genes having orthologs in mammals.

## Introduction

Dichlorodiphenyltrichloroethane (DDT) has gone from being a worldwide panacea of insect control and probably the most famous pesticide in modern history, to being a critical flashpoint of the modern environmental movement and becoming the most infamous pesticide of recent times. DDT was first used to control pest insect populations beginning in the 1940s, but instances of field resistance were observed among many species of insects, including *Drosophila melanogaster* (*Drosophila*) [1]. Subsequent deleterious side effects were observed in non-target mammalian and avian species, and were linked to the environmental persistence of this insecticide or its metabolites [2–5], ultimately leading to DDT being banned in most countries. However, DDT remains in industrial production due to its continued use for the control of mosquitoes that vector malaria; a niche where DDT is very effective [6].

DDT disrupts nervous system function in arthropods by affecting nerve cell plasma membrane permeability and causing paralysis [7]. Contrary to expectations, when selection pressures for DDT resistance were eliminated following bans on DDT use in many nations, the frequencies of resistance phenotypes remained high in many endemic pest insect populations. Persistence has been attributed to random genetic drift of alleles that have no fitness costs compared to susceptible counterparts [8]. Additionally, DDT resistance mechanisms can confer cross-resistance to pyrethroid [9, 10] and neonicotinoid insecticides [11], and may be a factor that contributes to maintenance of resistance alleles in the absence of a direct DDT selection [12].

The genetic basis of DDT resistance traits in the mosquito *Anopheles gambiae* involves the additive effects of two quantitative trait loci (QTL) [13]. Similarly, two QTL with major effects were mapped to the *para* sodium channel and the *CCEunk7* esterase genes in *Aedes aegypti*, along with minor QTL that implicated the role of 20 other detoxification enzymes [14]. Additionally, a significant amount of research on DDT resistance in *Drosophila* has focused on metabolic resistance [11, 12, 15–17].

DDT resistance in *Drosophila* is not a uniform phenotype, with varying levels of resistance observed across different *Drosophila* strains, and resistance can roughly be categorized into low-, medium- and high-level resistance [18]. Initial work on DDT by Crow [19] demonstrated the polygenic nature of DDT resistance, however, subsequent research singled out one low-level DDT resistant phenotype involving the *Rst(2)DDT* locus on chromosome two [20]. The chromosome region of *Rst(2)DDT* contains two cytochrome monooxygenease (P450) genes, *Cyp6g1* and *Cyp12d1*, that are over expressed in at least some DDT resistant strains [18, 21]. Transcription of *Cyp6g1* in DDT resistant *Drosophila* was up-regulated by an upstream insertion of the Accord transposon [22], and all subsequently described field resistant strains similarly show an over expression of *Cyp6g1* due to this Accord insertion [12].

In contrast, DDT resistance among laboratory strains involves over expression of multiple P450 genes in addition to *Cyp6g1* and *Cyp12d1* [23, 24]. Specifically, the laboratory selected strain *91-R* showed significant increases in expression of multiple cytochrome P450s and dozens of other genes. Furthermore, over expression of *Cyp6g1* in transgenic *Drosophila* with a susceptible genetic background failed to reconstitute high levels of DDT resistance [25] and reinforced the hypothesis that DDT resistance may be a multilocus trait in this species [17, 26]. Indeed, a recent toxicokinetic analysis of *91-R* revealed that oxidative P450s likely causes little direct metabolic resistance, but reduced cuticular penetration, increased reductive dechlorination, and enhanced excretion have been shown to play dominant roles [27].

With the advent of next generation sequencing (NGS) technologies, full-genome re-sequencing has become logistically feasible, and allows for ultra-fine resolution to map the genome location of mutations [28]. It is also a tool for genome-wide association studies (GWAS), population genomic [29] and phylogenomic studies [30]. GWAS using NGS-based re-sequencing approaches has been effectively applied to estimate genome variation within and between populations, and has identified genome regions that are associated with the expression of various traits [31–33]. GWAS in *Drosophila* are feasible due to a high-quality genome sequence assembly, gene models and tools for genome-wide molecular analyses [5, 34, 35].

The laboratory selected DDT resistant and control strains *91-R* and *91-C*, respectively, represent lines that have a common origin, have been kept side-by-side in the laboratory, yet while *91-C* received no DDT selection, *91-R* received intense DDT selection pressure for over 50 years. Using these resource populations, whole genome re-sequencing data were generated from pools of individuals from the *Drosophila* strains *91-R* and *91-C*, and applied to detect chromosome regions putatively affected by prolonged DDT selection. To the authors’ knowledge, analysis across the entire insect genome to identify regions influenced by selective sweeps in highly DDT resistant insects has not been previously performed, although there has been a study focused on insecticide resistant blow flies (*Lucilia cuprina*) examining selective sweeps around an individual gene [36] and some additional studies looking at signatures of selection around transposable elements [37, 38]. We tested the hypothesis that the expected usual gene suspects for DDT resistance would be detected, as opposed to the alternative that resistance is polygenic with many other genes impacting resistance. Elucidating these genetic and biochemical mechanisms associated with pesticide resistance evolution might lead to improved pest management strategies. Equally important, such information has the potential to further our understanding of how DDT impacts biological processes that are evolutionarily conserved between insects and mammals.

## Results

### Genome re-sequencing and data filtering

Read mapping with the Bowtie2 resulted in alignment of ≥ 98.5% of all 91-R and 91-C trimmed reads to the Drosophila reference genome release 5.7 resulted mean coverage depths of 63.6- and 62.0-X, respectively (S1 Table). The resulting mapping files were submitted to NCBI with an accession number SRP052046.

### Detection of selective sweeps in 91-R by mapping Pool-seq data

**Fig 1:**
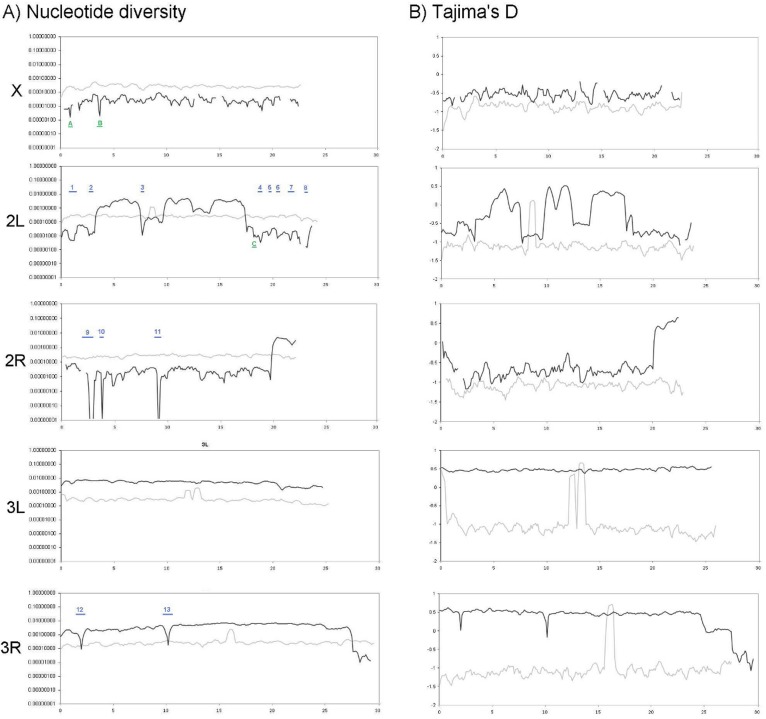
Estimates of (A) nucleotide diversity (pi; π) and (B) Tajima's D (*D*) across the chromosome arms of *Drosophila melanogaster* from the DDT resistant *91-R* strain (black line) and DDT susceptible *91-C* strain (grey line). Metrics obtained from 500-kb sliding windows with a step size of 100-kb. The genes identified to be associated with each of the major selection sweeps are as follows: (1) CG42329, (2) CG15394, (3) *NinaC*, (4) CG6453, (5) CG17568 and *Ref(2)P*, (6) *RtGEF*, (7) CG12050, CG8677, and *Dtr*, (8) CG31612, (9) *Dscam1*, (10) *Sut1*, (11) *MDR49*, (12) CG1041 and (13) CG31495. The genes identified to be associated with each of the minor selection sweeps are as follows: (A) *Cyp4g1*; (B) *AlstR*, *Mnt* and *Fd3F*; and (C) *Kon*.

Mean nucleotide diversity (*π*) and Tajima's D (*D*) estimates were made among major chromosome arms for mapped read data from strains *91-R* and *91-C* (Table 1). Both estimates were generally higher for *91-R* compared to *91-C* and were also ~10-fold lower on the X chromosome compared to autosomes (Table 1). The variation within sliding window estimates for *π* and *D* were also greater for 9*1-R* compared to *91-C*. Use of an arbitrary cutoff of a ≥ 100-fold reduction of *π* in a given 500-kb window compared to the mean *π* across the same chromosome resulted in the identification of 13 genome intervals in the *91-R* genome (Fig 1A). Strong evidence for directional selection was not found on chromosomes X or 3L. Reduced nucleotide diversity was used as a proxy for the identification of genome regions, which potentially show the effects of a selective sweep, where the size of these 13 genome regions ranged from 0.1 to 0.9-Mbp (Table 2). A second tier cutoff of a ≥ 90- but < 100-fold reduction of *π* in a given 500-kb window was used to identify putative genome regions affected to a lesser degree following chronic DDT exposure in *91-R*, and resulted in the identification of three additional genome intervals showing “minor” selective sweeps (Fig 1A). By comparison to estimates obtained across the genome of *91-C*, the effects of random genetic drift, as opposed to directional selection, could possibly be accounted for on the *91-R* genome. Decreases in the estimates of *D* along chromosome arms were also interpreted as being derived from the effects of directional selection in those corresponding genome regions. The calculated values of *D* along 500-kb windows of genomic sequence from *91-R* indicated the effects of negative section were detected in 2L and 3R, and that these genome regions corresponded to those also predicted to show significant reductions in *π* described above. Similar to the estimates of *π* described above, there were no intervals on chromosomes X or 3L that showed significant reductions in estimates of *D* in the *91-R* or *91-C* genome. In contrast, the estimates of *D* did appear to mirror those of *π* along 2L, 2R and 3R in *91-R* but not *91-C* (Fig 1B).

**Table 1:** Mean nucleotide diversity (pi; π) and Tajima's D (*D*) among chromosome arms for DDT resistant *91-R* and susceptible *91-C Drosophila melanogaster* strains.

	Mean *π*		Mean *D*	
Chromosome	*91-R*	*91-C*	*91-R*	*91-C*
X	0.0000287±0.0000173	0.0002626±0.000074	-0.5516±0.1172	-0.8784±0.1217
2L	0.0013216±0.001567	0. 0002698±0.000136	-1.1013±0.2103	-0.3383±0.4770
2R	0.0003640±0.001051	0.0002663±0.0000540	-0.5812±0.4047	-1.0866±0.1121
3L	0.0049182±0.001512	0.0003234 ±0.0002795	0.4785±0.0375	-1.0183±0.4060
3R	0.0037892±0.002277	0.0002899±0.0002664	0.4339±0.1668	-1.0558±0.3084

**Table 2:** Thirteen genomic intervals in *Drosophila melanogaster* strain *91-R* (ID 1 to 13) with nucleotide diversity estimated reduced ≥ 100-fold compared to respective chromosome means, and indicate putative genome regions under directional selection for survival when exposed to DDT.

ID	Chr	Start	Stop	*91-R π*	ID	Chr	Start	Stop	*91-R π*
1	2L	850,000	950,000	0.00000795	7	2L	21,050,000	21,150,000	0.00000622
1	2L	950,000	1,050,000	0.00000583	7	2L	21,150,000	21,250,000	0.00000841
1	2L	1,050,000	1,150,000	0.00000479	7	2L	21,250,000	21,350,000	0.00000265
1	2L	1,150,000	1,250,000	0.00000483	8	2L	21,750,000	21,850,000	0.00000172
1	2L	1,250,000	1,350,000	0.00000460	8	2L	21,850,000	21,950,000	0.00000148
1	2L	1,350,000	1,450,000	0.00000446	8	2L	21,950,000	22,050,000	0.00000602
2	2L	2,650,000	2,750,000	0.00000995	9	2R	2,750,000	2,850,000	0.00000150
3	2L	7,350,000	7,450,000	0.00001100	9	2R	2,850,000	2,950,000	0.00000001
4	2L	17,150,000	17,250,000	0.00000788	9	2R	2,950,000	3,050,000	0.00000001
4	2L	17,250,000	17,350,000	0.00001210	9	2R	3,050,000	3,150,000	0.00000001
4	2L	17,350,000	17,450,000	0.00000843	9	2R	3,150,000	3,250,000	0.00000001
4	2L	17,450,000	17,550,000	0.00000823	10	2R	3,950,000	4,050,000	0.00000001
4	2L	17,550,000	17,650,000	0.00001138	11	2R	8,850,000	8,950,000	0.00000001
4	2L	17,650,000	17,750,000	0.00000750	11	2R	8,950,000	9,050,000	0.00000001
4	2L	17,750,000	17,850,000	0.00000328	12	3R	1,850,000	1,950,000	0.00074201
4	2L	17,850,000	17,950,000	0.00000563	12	3R	1,950,000	2,050,000	0.00045369
5	2L	19,250,000	19,350,000	0.00000650	12	3R	2,050,000	2,150,000	0.00008574
5	2L	19,350,000	19,450,000	0.00000918	12	3R	2,150,000	2,250,000	0.00043772
5	2L	19,450,000	19,550,000	0.00000947	12	3R	2,250,000	2,350,000	0.00096688
6	2L	20,350,000	20,450,000	0.00000441	13	3R	9,550,000	9,650,000	0.00078830
6	2L	20,450,000	20,550,000	0.00000653	13	3R	9,650,000	9,750,000	0.00017920

Each interval (ID number) is represented by ≥ 1 100-kb sliding window that spans a putative selective sweep identified from estimates of nucleotide diversity (pi; *π*) (Fig 1). Due to either the size of the interval > 1 sliding window region was identified in selective sweeps for all but intervals (IDs) 2, 3, and 10.

### Identification and annotation of candidate genes in selective sweeps

The location of 67,835 *91-R* and 58,376 *91-C* single nucleotide polymorphisms (SNPs) were mapped to all gene-coding regions on chromosomes 2L, 2R and 3R (S2, S3 and S4 Tables). Mapping was not conducted on chromosomes X or 3L due to lack of selective sweeps detected on those chromosome regions. The genes in each nucleotide interval identified within selective sweeps from the *91-R* genome were interrogated for further evidence of directional selection using excess of nucleotide mutations that cause amino acid changes. Specifically, the ratios of the rate non-synonymous to synonymous mutation (K_A_/K_S_) were calculated for gene in each selective sweep, and those with a K_A_/K_S_ > 1 were considered candidates genes with evidence of DDT selection (S5 Table). Functional and mutant annotations for each gene were retrieved from Flybase.org using gene symbol in a keyword search and distribution of tissues in which transcripts for each gene have been previously identified were found in Flybase.org. Derived protein sequences were obtained for all genes putatively involved in DDT resistance in *91-R* (S6 Table), and when possible the functional domains were mapped with respect to the site of amino acid changes (S7 Table).

## Discussion

In the current study we used a whole genome approach to detect nucleotide signatures of directional selection in the *91-R* population that resulted following chronic DDT exposure. Specifically, the effects of DDT selection on localized regions of the *91-R* genome were measured by reductions in nucleotide diversity and corresponding estimates of directional selection using Tajima's *D*. The implication of individual genes in DDT resistance was obscured using this approach since genetic hitchhiking of flanking genes and genome regions occurs during selection due to limits on recombination to reduce the size of haplotype blocks [39, 40]. Thus, selective sweeps in *91-R* have resulted in the near fixation of nucleotide sequence of the causal genetic factor(s) that are directly involved in the genes giving rise to this DDT resistant phenotype, as well as genes in proximity. It is important to note that some gene(s) located within some of the selective sweeps may appear associated with the resistant phenotype in *91-R*, but may have been carried to fixation due to physical genetic linkage to gene(s) with major effect due to genetic hitchhiking.

Overall, genes within the 13 major (Fig 1 and S6 Table) and three minor effect regions (Fig 1 and S6 Table) identified in *91-R* by selective sweeps do not correspond to the genes that have been previously associated with DDT resistance using gene expression analyses or identified in pesticide resistant *Drosophila* populations. The only exception is gene *NinaC*, which has predicted kinase activity related to sensory transduction/vision, and is over-expressed in DDT resistant strains [26]. Similarly, four of the genes located in genome regions of *91-R* affected by selective sweeps, *Dscam1*, *Dtr*, *RtGEF*, and CG6453, have previously been implicated in synaptic development and/or function [41–44]. Additionally, the candidate genes of *91-R* affected by selective sweeps may also be involved in regulation of cellular growth (CG6453) [44], as well as genes that are involved in cellular communication and signal transduction cascades (S4 Table). Although implication of these mutant alleles in DDT resistance can be rationalized, additional functional studies are required to deduce individual roles as well as the effects of the non-synonymous changes in *91-R* on subsequent protein function and resulting phenotype. Additionally, the nucleotide diversity (pi; *π*) and D in the sliding window figure indicated that the control (*91-C*) strain showed no evidence for reduced variation or coding sequences (CDS) selection, in that sliding windows are fairly uniform across the chromosome—with exception of certain regions likely representing those near centromeres.

The genes CG17568, *ref(2)P*, CG8677, and CG31612 located in selective sweeps five, seven and eight, respectively, contain Zn-finger DNA binding motifs, which could suggest the resultant proteins have potential roles as transcription factors. Since *trans*-regulatory control of transcription by soluble transcription factors often occurs at *cis*-promoter or enhancer elements by way of Zn-finger mediated protein-DNA interactions, mutations in transcription factors that affect DNA-protein or protein-protein interactions at the promoter or with enhancer elements can cause changes in expression at physically unlinked genes. Therefore, genes in an interconnected gene regulatory pathway may show a coordinated response to transcription factor mutations. It is conceivable (but speculative) that the mutant transcription factor alleles in *91-R* might be involved in the gene regulatory networks which lead to up-regulation of the transcripts in *91-R* described by Pedra et al. [17], a hypothesis that remains to be tested. In a broader context, the basal cause of apparent incongruent results obtained from gene expression and genetic mapping/phylogenomic studies may be rooted in the effect that genes in QTL intervals/selective sweeps have upon gene networks. This hypothesis might also suggest that system approaches may yield greater insight into the genetic and genomic basis of insecticide resistance traits.

In the three genome regions showing less major effects of selection at a ≥ 90-fold reduction in nucleotide diversity cutoff, (A, B and C in Fig 1 and S2 Table), the candidate genes were associated with the nervous system and only one was a P450, *Cyp4g1*. The *Cyp4g1* protein is known to be associated with hydrocarbon production, converting long-chained aldehydes to long-chained hydrocarbons in oenocytes in the epidermis of *Drosophila*, that are then in turn transported to the waxy layer of the epicuticle [45]. Additionally, Strycharz et al. [27] recently demonstrated that *91-R* had higher quantities of cuticular hydrocarbons, visible changes in the cuticle (via electron microscopy), and that reduced penetration is an important component of DDT resistance in *91-R*. Although work by Waters et al. [46] suggested no difference in expression levels of *Cyp4g1* between *91-C* and *91-R*, it is not currently known if differential expression of the *Cyp4g1* protein might be localized near the cuticle of the insect or if structural changes in *Cyp4g1* may play some role in resistance.

Although ATP-binding cassette (ABC) transporters have previously been associated with DDT resistance in *Drosophila*, the selective sweeps analysis has shed new light on an additional candidate DDT resistance gene known as *MDR49*. Strycharz et al. [27] previously compared the transcription levels of the ATP-binding cassette transporters *MDR49*, *MDR50* and *MDR65* and *MRP1* in *91-R* versus *Canton-S* strains. Interestingly, *MDR50*, *MDR65* and *MRP1* were over-expressed in *91-R* whereas *MDR49* was not. RNAi knockdown of *MDR50*, *MDR65* and *MRP1* in DDT resistant flies results in increased sensitivity to DDT, however, knockdown of *MDR49* had no effect [47]. Such aforementioned experimental approaches would only detect the putative role of differential transcription in DDT resistance and not structural changes in the protein that may lead to DDT resistance. Thus, the amino changes that we observed in *MDR49* that may play a role in DDT resistance, if any, remain to be determined.

The other genes co-occurring with selective sweeps included: *Dscam1*, *NinaC*, CG6453, CG17568, *Ref(2)P*, *RtGEF*, CG12050, CG8677, *Dtr*, CG31612, *Sut1*, CG1041, and CG31495. Several of these aforementioned genes located within the identified selective sweeps contain known or suspected orthologs in other animals, including mammals, which could be useful in further investigation to better understand their potential links to DDT resistance. For example, some of these genes show plausible linkages to phenotypic resistance to DDT, however, based on their roles in mammals in biological processes known to be impacted by DDT exposure. For example, *Dscam1* is associated with psychomotor retardation, and DDT (or more specifically the DDE byproduct) has been linked with retarded psychomotor development in humans exposed in the first trimester [48, 49]. *NinaC* is a retinal specific gene that codes for two photoreceptor cell specific proteins in *Drosophila* [50, 51], and mutations in *NinaC* were shown to cause light- and age-dependent retinal degeneration in *Drosophila* [51]. In human studies, Kamel et al. [52] found a dose-response relationship between exposure to organochlorides, such as DDT, and the risk of retinal degeneration. Male sterility in *Drosophila* results when *Ref(2)P* gene expression is absent in the testes, which suggested that the *Ref(2)P* gene expression is required for successful reproduction [53]. Although a somewhat controversial topic in the literature, there have been studies indicating that environmental pollutants (such as DDT) have links to male infertility [54, 55] in both humans [54] and rats [55]. The role these genes may play in high level DDT resistance remains to be determined. Additionally, it remains to be determined if some of these evolutionarily conserved, between insects and mammals, candidate genes may also provide insights into the impact of DDT exposure in mammalian systems, as has been done previously using *Drosophila* and human diseases [56–60].

Interestingly, we did not observe many of the genes typically associated with pesticide resistance [19, 61–65, 68–76]. Many initial publications reported that moderate to high level DDT resistance is thought to be polygenic with multiple genome regions contributing to this phenotype [19, 63, 64]. Previous researchers have identified loci on the second chromosome involved in DDT resistance [20, 65], whereas chromosomes X and three are thought to have some slight impact on the DDT resistance phenotype [64, 65]. The uniform reduction in estimates of *π* across the X-chromosome of 91-C and 91-C, were not surprising due to the effective 3/4 population size of the X-chromosome as compared to autosomes [66], which affects the rate at which chromosomal loci may become fixed by random genetic drift or influenced by selection [77]. The affects of random fixation by random genetic drift on the X-chromosome may also have been exacerbated by the relatively small number of individuals in 91-C and 91-R laboratory populations [67]. An increasing amount of research has focused on the single *Rst(2)DDT* locus [20, 68–71], whereby over-transcription of the cytochrome P450 *Cyp6g1* located within the *Rst(2)DDT* region was suggested to be both necessary and sufficient for DDT resistance [25]. Daborn et al. [25] essentially proposed that resistance to DDT was monogenic, at least in *Drosophila* strains with low-level DDT resistance. The *Rst(2)DDT* locus maps to the second chromosome between the genes *cinnabar* (*cn*; location 2R:3,670,302–3,672,711) and *vestigial* (*vg*; 2R:8,772,137–8,786,890) [11, 21, 72, 73]. Although our current dataset is not from a low-level resistant strain, this genome interval in *91-R* where *Cyp6g1* exists is located between, and not within, the *91-R* selective sweeps labeled 9 and 10 (Fig 1). Interestingly *Cyp6g1* is over-expressed in the 91-R strain [17, 21, 78]. Thus, our analysis suggests that *Rst(2)DDT* is not a major factor, or potentially even involved, in the DDT resistance phenotype in *91-R*, and agrees with prior results which showed that DDT resistance could be maintained in *Drosophila* strains that did not show high *Cyp6g1* transcript levels derived from at the *Rst(2)DDT* locus [74]. The current results are also in agreement with the conclusion by Strycharz et al. [27] that metabolic resistance, particularly P450-based resistance, plays a negligible role in the overall DDT resistance phenotype in *91-R*. In addition to *Cyp6g1*, expression of *Cyp12d1* was implicated in being differentially expressed in DDT-resistant fly strains [18] as were non-synonymous coding sequence mutations *Cyp6a2* [75] and *para* [76]. However, none of these genes occurred in any of the selective sweeps identified in our current experiments.

DDT resistance, however, is not a single phenotype, but varies among strains from low- (*e*.*g*., *Hikone-R*), to moderate- (*e*.*g*., *Wisconsin*), to high-levels (*e*.*g*., *91-R*) [18]. Based on microarray analysis, moderate- to high-level resistant phenotypes appeared to result from the effect of multiple differentially-regulated genes. Specifically, using microarray data, Pedra et al. [17] observed that numerous genes were over-transcribed in the *91-R* strain including cytochrome P450s, glutahione S transferases, and a set of additional genes. Comparative analysis of microarray data from the *Wisconsin* and *91-R* strains showed that multiple genes were differentially expressed, and that these genes were more numerous in the more highly resistant strain than the moderately resistant strain [17, 79]. A proteomic analysis also revealed that proteins associated with energy metabolism were differentially expressed in two DDT resistant as compared with a susceptible strain [26]. These combined observations suggest that at least moderate- to high-level DDT resistance may involve complex molecular interactions, and this might be consistent with a resistant phenotype that results from the effects of multiple genes. These results also suggest that several different genetic mechanisms may result in DDT resistant phenotypes, and that increasing levels of DDT resistance may be additive with an increasing number of genes involved, a hypothesis that remains to be tested. However, these results do not rule out the fact that low-level DDT resistant strains, taken directly from the field, may have fewer, i.e., monogenic, molecular mechanisms of resistance such as overly transcribed *Cyp6g1* [78].

Certainly, the current data and analysis could be of potential importance to those insecticides where DDT resistance has been shown to confer cross-resistance to other types of insecticides, such as imidacloprid in *Drosophila* and pyrethroids in *A*. *aegypti* [11, 80]. Of greater practical importance, however, this general GWAS approach could be applied to other insect species currently being controlled by other pesticides, in order to understand the evolution of resistance in “real time” (*i*.*e*., follow field populations through generations of selection). A number of other insect genomes have been sequenced since the *Drosophila* genome was published in 2000 and has allowed for the study of insecticide resistance at the molecular level for a variety of species, such as in *Anopheles gambiae* [34, 81]. Although the *91-C* and *91-R* fly lines provide a unique system where selection has occurred for over half a century, there exist multiple *Aedes aegypti* laboratory strains, including strains selected for insecticide resistance to permethrin, where similar studies could be performed to identify structural mutations across the genome [82].

The discoveries of novel resistance mechanisms from such studies could help lead to new target genes and the development of novel control methods for these resistant species [83, 84]. This work also highlights that selection with DDT may result in the selection for novel mutations, potentially with some or many of these being associated with or directly involved in DDT resistance. Additional studies are required to validate the role of genes in each predicted selective sweep in DDT resistance by verifying the functional consequence of amino acid changes on protein structures and potential impact on the *91-R* resistant phenotype. Of greatest importance, this study highlights the need for selective sweep analyses in pesticide resistant insect populations in order to identify potential candidate resistance traits. Further molecular examination of individual genes and a more detailed analysis of the specific effects of the structural changes within the insects are crucial to better understanding resistance, something beyond the scope of the current project.

## Materials and Methods

### Genome re-sequencing and data filtering

Dr. Ranjan Ganguly of the University of Tennessee-Knoxville provided the DDT resistant and susceptible *Drosophila* strains, respectively *91-R* and *91-C* [85]. For detailed description of fly line maintenance, re-sequencing, and data filtering, please see Steele et al. [86].

### Detection of selective sweeps in 91R by mapping Pool-seq data

#### Estimates of nucleotide diversity

A pooled sequencing approach (Pool-seq) was used to compare the nucleotide variance at all positions across the *Drosophila* strains *91-C* and *91-R* genomes, with the goal of identifying putative regions of reduced nucleotide diversity in *91-R* that putatively correspond to regions affected by directional selection (selective sweeps) [87, 88]. To accomplish this, quality score trimmed reads from *91-R* and *91-C* libraries were aligned separately to the *Drosophila* genome assembly release 5.7 (file dmel-all-chromosomee-r5.7.fasta downloaded from Flybase.org) using Bowtie2 with parameters-l 100-n 0.01-o 2-e 12-d 12 [89]. Bowtie2 output in SAM format was converted to a sorted BAM file and synchronized with the SamTools mpileup command [90]. The BAM files have been deposited at NCBI with accession number of SRP052046. Nucleotide diversity (*π*) estimates were calculated across the alignments for *91-C* and *91-R* data in 500-kb sliding windows with a step size of 100 kb using the Perl script Variance-sliding.pl from the PoPoolation Package [91] with a minimum coverage = 2, maximum coverage = 75, and minimum quality = 25. The maximum coverage was restricted to 2-times the mean read depth to reduce the incidence of SNP detection within repetitive DNAs. Windows where genome regions lacked any SNPs were reported as "na", and were counted as missing data such that gaps were present in the resulting plots. Regions of the genome with evidence of putative selective sweeps were identified using an arbitrary cutoff of a *π* 100-fold reduction of *π* within a window compared to the mean *π* across the same chromosome. Mapping data to the Y chromosome, mitochondrial genome and chromosome four were excluded from analyses.

#### Estimates of Tajima's D

Tajima's D (*D*) estimates were independently obtained for alignments of *91-R* and *91-C* reads using the script Variance-sliding.pl from the PoPoolation Package [91] using parameters identical to those used to estimate *π*, except a uniform coverage of 30 was used to account for heterogeneous expectations of *D* since the measure is dependent upon the number of chromosomes (coverage depth). The *D* estimates provided by PoPoolation do not take into account the potential of multiple sampling, thus a negative bias is likely among resulting output but was expected to be equally represented across the genome such that general inferences of the effects of directional and balancing selection in genome regions could be made [92]. Resulting estimates of *D* for *91-R* and *91-C* were plotted along the lengths of each chromosome arm. Gene-by-gene estimates of *D* were also made using the script Variance-at-position.pl (measure = *D*), with the file Drosophila_melanogaster_BDGP5.72.gtf used to define gene coding intervals. Sampling of the data also used a uniform depth of 30 across each gene.

### Identification and annotation of candidate genes in selective sweeps

Genes in genome intervals with reduced estimates of *π* and *D* were considered candidate DDT resistance genes, but the pools were narrowed using an _N_/*π*_S_ cutoff > 1.0. Specifically, nucleotide diversity at synonymous (*π*_S_) and nonsynonymous codon positions (*π*_N_) was estimated for all genes in *91-C* and *91-R* alignment data using the Perl script syn-nonsyn-at-position.pl (measure = pi), where gene coding positions were defined in the file Drosophila_melanogaster_BDGP5.72.gtf (http://www.ensembl.org/info/data/ftp/index.html). SNPs predicted with a minimum count of 4, minimum coverage of 8, and a maximum coverage of 75 for both *91-C* and *91-R* datasets. Gene coding regions that lacked synonymous and/or non-synonymous mutations were excluded from subsequent calculations of *π*_N_/ *π*_S_, and the ratio was used to predict genes with an excess of non-synonymous site mutation (*π*_N_/ *π*_S_ > 1.0).

Instances in each putative selective sweep where alleles had become fixed in strain *91-R* but remained variable in the *91-C* genome were identified manually. Functional gene annotation data were retrieved for candidate genes from FlyBase (http://flybase.org/) using a keyword search gene symbol. Derived protein coding sequences were constructed using predicted non-synonymous mutation predictions made from *91-R* and *91-C* SNP data, and used as a query against the NCBI nr protein database using the blastp algorithm (hit cutoff set for *E*-values ≤ 10^–20^). Conserved functional protein domains were identified by searches against the Conserved Domain Database (CDD) [93], and used to annotate the derived proteins from candidate DDT resistance genes from *91-R*. These variable amino acids positions in *91-R* were plotted with respect to protein functional domains (when known).

## Supporting Information

S1 Table Mapping statistics for *Drosophila melanogaster 91-R* and *91-C* specific read libraries to the reference genome release 5.7 using Bowtie2 [89]. All reads reported in millions.
(DOCX)

S2 Table Locations of those 67,835 *91-R* and 58,376 *91-C* single nucleotide polymorphisms (SNPs), located on chromosome 2L, mapped to all gene-coding regions on the chromosome. (TXT)

S3 Table Locations of those 67,835 *91-R* and 58,376 *91-C* single nucleotide polymorphisms (SNPs), located on chromosome 2R, mapped to all gene-coding regions on the chromosome. (TXT)

S4 Table Locations of those 67,835 *91-R* and 58,376 *91-C* single nucleotide polymorphisms (SNPs), located on chromosome 3R, mapped to all gene-coding regions on the chromosome. (TXT)

S5 Table Genes in the genome of the *Drosophila melanogaster 91-R* strain that are within regions putatively affected to a lesser extent by selective sweeps caused by fixation of DDT resistant traits (please see Fig 1). Expression in adult head (hd), brain (br), malpigian tubules (mt), central nervous system (cns) and embryonic tissues (emb) are shown as indicated in FlyBase.org. These genome regions did not surpass the arbitrary cutoff of 100-fold reductions in nucleotide diversity, but did shown an estimated ≥90-fold decreases when compared to the average across respective chromosomes.
(DOCX)

S6 Table Genes in the genome of the *Drosophila melanogaster 91-R* strain that are within regions putatively affected by selective sweeps caused by fixation of DDT resistant traits (please see Fig 1). Expression in adult head (hd), brain (br), malpigian tubules (mt), central nervous system (cns) and reproductive tissues (rt; testis and/or ovaries) are shown as indicated in FlyBase.org.
(DOCX)

S7 Table Non-synonymous nucleotide and associated amino acid changes in candidate genes identified within thirteen major genome regions in the *91-R Drosophila melanogaster* strain showing influence of a selective sweep. (DOCX)
