# Supplementary material for: Selective Sweep Analysis in the Genomes of the 91-R and 91-C Drosophila melanogaster Strains Reveals Few of the ‘Usual Suspects’ in Dichlorodiphenyltrichloroethane (DDT) Resistance
Source: PLoS One. 2015 Mar 31;10(3):e0123066. doi: 10.1371/journal.pone.0123066 (PMC4380341; doi:10.1371/journal.pone.0123066)
Supplement: S1 Table — All reads reported in millions. (DOCX) [file pone.0123066.s001.docx]

**Supplemental Table S1**: Mapping statistics for *Drosophila melanogaster* *91-R* and *91-C* specific read libraries to the reference genome v.7.1 using Bowtie2 (Langmead and Salzberg 2012). All reads reported in millions.

| Strain | SRA submission | Total | Processed | Unique | > 1 | unmapped |
| --- | --- | --- | --- | --- | --- | --- |
| 91-R | SRP041176 | 148.1 | 107.4 | 70.9 | 25.6 | 10.9 |
| 91-C | SRP041176 | 139.3 | 104.4 | 71.8 | 26.2 | 6.4 |
