# Supplementary material for: Selective Sweep Analysis in the Genomes of the 91-R and 91-C Drosophila melanogaster Strains Reveals Few of the ‘Usual Suspects’ in Dichlorodiphenyltrichloroethane (DDT) Resistance
Source: PLoS One. 2015 Mar 31;10(3):e0123066. doi: 10.1371/journal.pone.0123066 (PMC4380341; doi:10.1371/journal.pone.0123066)
Supplement: S5 Table — Expression in adult head (hd), brain (br), malpigian tubules (mt), central nervous system (cns) and embryonic tissues (emb) are shown as indicated in FlyBase.org. These genome regions did not surpass the arbitrary cutoff of 100-fold reductions in nucleotide diversity, but did shown an estimated ≥90-fold decreases when compared to the average across respective chromosomes. (DOCX) [file pone.0123066.s005.docx]

**Supplementary Table S5**. Genes in the genome of the *Drosophila melanogaster* *91-R* strain that are within regions putatively affected to a lesser extent by selective sweeps caused by fixation of DDT resistant traits (please see Figure 1). Expression in adult head (hd), brain (br), malpigian tubules (mt), central nervous system (cns) and embryonic tissues (emb) are shown as indicated in FlyBase.org. These genome regions did not surpass the arbitrary cutoff of 100-fold reductions in nucleotide diversity, but did shown an estimated ≥90-fold decreases when compared to the average across respective chromosomes.

|  |  |  |  |  | Tissue specific transcription | | | | |
| --- | --- | --- | --- | --- | --- | --- | --- | --- | --- |
| ID | Genome interval | Flybase ID | Gene | Functional annotation | hd | br | mt | emb |  |
| A | X: 361,090.. 363,368 [-] | FBgn0010019 | *Cyp4g1** | Cytochrome P450 |  |  |  | E |  |
| B | X: 3,468,569..3,560,757 [+] | FBgn0028961 | *AlstR*† | neuropeptide signaling pathway |  |  |  | X |  |
| B | X: 3,565,834..3,597,318 [+] | FBgn0023215 | *Mnt*‡ | neuron projection morphogenesis |  |  |  | X |  |
| B | X: 3,696,349..3,705,073 [+] | FBgn0264954 | *Fd3F*§ | forkhead domain 3F; transcription factor; dendrite morphogenesis |  |  |  | X |  |
| C | 2L:18,487,747..18,503,978 [-] | FBgn0032683 | *Kon*¶ | Neurogenesis |  |  |  | X |  |

* Waters, L.C., Zelhof, A.C., Shaw, B.J., Ch'ang, L.Y. (1992). Possible involvement of the long terminal repeat of transposable element 17.6 in regulating expression of an insecticide resistance-associated P450 gene in *Drosophila*.  Proc. Natl. Acad. Sci. U.S.A. 89: 4855-4859.

† Hewes, R.S., Taghert, P.H. (2001). Neuropeptides and neuropeptide receptors in the *Drosophila melanogaster* genome.  Genome Res. 11(6): 1126-1142.

‡ Sepp, K.J., Hong, P., Lizarraga, S.B., Liu, J.S., Mejia, L.A., Walsh, C.A., Perrimon, N. (2008). Identification of neural outgrowth genes using genome-wide RNAi.  PLoS Genet. 4(7): e1000111.

§ Lee, H.H., Frasch, M. (2004). Survey of forkhead domain encoding genes in the Drosophila genome: classification and embryonic expression patterns. Dev. Dyn. 229(2): 357-366.

¶ Neumüller, R.A., Richter, C., Fischer, A., Novatchkova, M., Neumüller, K.G., Knoblich, J.A. (2011). Genome-wide analysis of self-renewal in *Drosophila* neural stem cells by transgenic RNAi. Cell Stem Cell. 8(5): 580-593.
