# Supplementary material for: Selective Sweep Analysis in the Genomes of the 91-R and 91-C Drosophila melanogaster Strains Reveals Few of the ‘Usual Suspects’ in Dichlorodiphenyltrichloroethane (DDT) Resistance
Source: PLoS One. 2015 Mar 31;10(3):e0123066. doi: 10.1371/journal.pone.0123066 (PMC4380341; doi:10.1371/journal.pone.0123066)
Supplement: S6 Table — Expression in adult head (hd), brain (br), malpigian tubules (mt), central nervous system (cns) and reproductive tissues (rt; testis and/or ovaries) are shown as indicated in FlyBase.org. (DOCX) [file pone.0123066.s006.docx]

**Supplemental Table S6:** Genes in the genome of the *Drosophila melanogaster* *91-R* strain that are within regions putatively affected by selective sweeps caused by fixation of DDT resistant traits (please see Figure 1). Expression in adult head (hd), brain (br), malpigian tubules (mt), central nervous system (cns) and reproductive tissues (rt; testis and/or ovaries) are shown as indicated in FlyBase.org.

|  |  |  |  |  | Known tissue specific transcription | | | | |
| --- | --- | --- | --- | --- | --- | --- | --- | --- | --- |
| ID | Genome interval | Flybase ID | Gene | Functional annotation | hd | br | mt | cns | Rt |
| 1 | 2L:1,219,293..1,229,802 [+] | FBgn0259229 | CG42329 | Acytltransferase; NFR6 homolog; transmembrane proteins function to transport fluoxetine across the hypodermal barrier to the inside of the animal, acts on neuromuscular targets to induce muscle contraction. Role in regulation of membrane transport |  |  | X |  |  |
| 2 | 2L:2,591,869..2,592,773 [+] | FBgn0250835 | CG15394 | Unknown | X | X |  |  |  |
| 3 | 2L:7,377,702..7,384,344 [+] | FBgn0002938 | *NinaC* | Serine/threonine-protein kinase; P-loop NTP hydrolase; motor activity | X |  |  |  |  |
| 4 | 2L:17,481,988..17,484,307 [-] | FBgn0032643 | CG6453 | Glucosidase 2 subunit beta; Involved in cellular lateral inhibition | X |  | X | X |  |
| 5 | 2L:19,179,749..19,181,702 [-] | FBgn0032763 | CG17568 | Zinc finger (C2H2) domain containing protein; DNA binding; |  |  |  |  | X |
| 5 | 2L:19,542,468..19,545,548 [+] | FBgn0032763 | *Ref(2)P* | Zinc finger ZZ-type (CX2C); Phox and Bem1p domain signaling protein; |  |  |  | X | X |
| 6 | 2L:20,350,434..20,365,254 [+] | FBgn0015803 | *RtGEF* | GTPase; Dbl homology (DH) domain |  |  |  |  |  |
| 7 | 2L: 21,094,966..21,102,429 [-] | FBgn0032915 | CG12050 | Unknown function; predicted WD40-repeat-containing domain; protein-protein interactions; signal transduction and transcription regulation to cell cycle control and apoptosis; |  |  |  | X | X |
| 7 | 2L:21,221,312..21,231,694 [+] | FBgn0026577 | CG8677 | Zn finger PHD-type (C4HC3); DNA binding homeobox and Transcription factors domain |  |  |  |  |  |
| 7 | 2L:21,361,216..21,367,288 [+] | FBgn0023090 | *Dtr* | Defective transmitter release |  |  |  |  | X |
| 8 | 2L:21,762,314..21,795,447 [-] | FBgn0051612 | CG31612 | Zinc finger (C2H2) domain containing protein; DNA binding |  |  |  |  |  |
| 9 | 2R:3,205,429..3,269,404 [-] | FBgn0033159 | *Dscam1* | Axon guidance receptor activity | X |  |  | X |  |
| 10 | 2R:3,970,481..3,974,656 [+] | FBgn0028563 | *Sut1* | Glucose transmembrane transporter activity |  |  |  | X |  |
| 11 | 2R:8,827,828..8,833,852 [-] | FBgn0004512 | *MDR49* | ACB transporter; P-loop NTP hydrolase | X |  |  | X |  |
| 12 | 3R: 2,196,748..2,201,662 [-] | FBgn0037440 | CG1041 | Acyltransferase ChoActase/COT/CPT | X |  |  | X |  |
| 13 | 3R: 9,663,962..9,665,656 [-] | FBgn0051495 | CG31495 | ATPase AAA-type core; P-loop NTP hydrolase |  |  |  |  |  |
