# Supplementary material for: Selective Sweep Analysis in the Genomes of the 91-R and 91-C Drosophila melanogaster Strains Reveals Few of the ‘Usual Suspects’ in Dichlorodiphenyltrichloroethane (DDT) Resistance
Source: PLoS One. 2015 Mar 31;10(3):e0123066. doi: 10.1371/journal.pone.0123066 (PMC4380341; doi:10.1371/journal.pone.0123066)
Supplement: S7 Table — (DOCX) [file pone.0123066.s007.docx]

**Supplemental Table S7.** Non-synonymous nucleotide and associated amino acid changes in candidate genes identified within thirteen major genome regions in the *91-R Drosophila melanogaster* strain showing influence of a selective sweep.

| Chr | Gene | Nucleotide changes | Amino acid changes |
| --- | --- | --- | --- |
| 2L | CG42329 | 7, G to C; 55, A to G; 175, G to C; | 3, V to L; 19, T to A; 59, E to Q; |
|  |  | 601, C to T; 667, T to A; 878, T to C; | 201, L to F; 223, S to C; 293, M to T; |
|  |  | 1384, G to C; 1534, G to A; 1736, G to C; | 462, G to R; 512, V to M; 579, S to T; |
|  |  | 1997, C to A; 2068, G to A | 666, P to Q; 690, D to N |
| 2L | CG15394 | 46, G to C; 88, A to C; 97, T to A; | 16, G to R; 30, M to L; 33, L to M |
| 2L | *NinaC* | 167, C to G; 4344, G to C | 56, S to C; 1448, K to N; |
| 2L | CG6453 | 7, T to C; 74, G to A; 328, C to T; | 3, S to G; 25, S to F; 110, G to S; |
|  |  | 478, T to A | 160, T to S |
| 2L | CG17568 | 415, C to T; 478, T to C; 479, G to T; | 139, E to K; 160, T to A; 160, T to K; |
|  |  | 665, G to A; 719, T to G; 742, G to T; | 222, A to V; 240, D to A; 248, Q to K; |
|  |  | 781, T to C | 261, T to A |
| 2L | CG10043 | 466, C to T; 2215, A to G; 2528, G to A; | 156, P to S; 739, T to A; 843, R to H; |
|  |  | 2779, G to A; 2824, G to A; 4058, G to A | 927, A to T; 942, D to N; 1353, S to N |
| 2L | CG12050 | 446, T to G; 662, G to C; 688, C to T | 149, K to T; 221, S to C; 230, D to N |
|  |  | 1081, A to G; 3052, C to T | 361, S to P; 1018, E to K |
| 2L | CG8677 | 185, A to G; 380, G to A; 5132, C to T; | 62, N to S; 127, R to K; 1711, A to V; |
|  |  | 8036, A to C | 2679, H to P |
| 2L | CG31623 | 1096, A to G; 1186, A to G; 1647, G to T; | 366, I to V; 396, M to V; 549, L to F; |
|  |  | 2135, T to G; 2296, T to A; 2383, G to A; | 712, F to C; 766, Y to N; 795, D to N |
|  |  | 2462, T to A; 3225, G to T; 3805, C to T; | 821, F to Y; 1075, Q to H; 1269, R to C; |
|  |  | 3805, C to T; 4297, G to A | 1269, R to C; 1433, D to N |
| 2L | CG31612 | 2672, T to C; 2674, C to T | 891, N to S; 892, G to S |
| 2R | *MDR49* | 5, A to G; 1121, G to A; 1162, T to A; | 2, V to A; 374, T to I; 388, M to L; |
|  |  | 2854, T to C | 952, I to V |
| 3R | CG1041 | 66, T to G; 399, A to C; 839, T to A; | 22, E to D; 133, N to K; 280, Y to F; |
|  |  | 974, A to G | 325, I to T |
| 3R | CG31495 | 63, G to T; 356, G to A; 398, T to A | 21, N to K; 119, S to F; 133, K to M |
|  |  | 641, T to A; 647, G to A; 745, C to G | 214, K to M; 216, T to M; 249, V to L |
